# 5Z-7-Oxozeanol Isolated from the Fungus *Curvularia* sp. MDCW-1060 Inhibits the Proliferation of MDA-MB-231 Cells via the PI3K-Akt and MAPK Pathways

**DOI:** 10.3390/md23110414

**Published:** 2025-10-23

**Authors:** Hong Zhang, Jianjian Wang, Chang Xu, Kai Liu, Jufang Xie, Zhoucheng He, Yonghong Liu, Cong Wang, Xinjian Qu

**Affiliations:** 1 Institute of Marine Drugs/Guangxi Key Laboratory of Marine Drugs/Guangxi University Engineering Research Center of High-Efficient Utilization of Marine Traditional Chinese Medicine Resources, Guangxi University of Chinese Medicine, Nanning 530200, China; 2 Key Laboratory of Chemistry and Engineering of Forest Products/State Ethnic Affairs Commission; Guangxi Key Laboratory of Chemistry and Engineering of Forest Products/Engineering Research Center of Low-Carbon and High-Quality Utilization of Forest Biomass, University of Guangxi/School of Chemistry and Chemical Engineering/Guangxi Minzu University, Nanning 530006, China

**Keywords:** 5Z-7-Oxozeaenol, marine natural products, anti-proliferation, MDA-MB-231 cells, RNA-Seq

## Abstract

The discovery of novel marine natural products and their sustainable application continue to be vital focuses in marine biological research. The aim of this study is to investigate the inhibitory effect of the compound 5Z-7-Oxozeaenol isolated from the fungus *Curvularia* sp. MDCW-1060 on the proliferation of MDA-MB-231 cells and its molecular mechanism. A series of functional assays, including 3-(4,5-Dimethylthiazol-2-yl)-2,5-diphenyltetrazolium bromide (MTT), flow cytometry, Transwell migration, and colony formation, were employed to evaluate the effects of 5Z-7-Oxozeaenol on cellular viability, apoptosis, migration, and clonogenicity. The RNA sequencing (RNA-seq) coupled with bioinformatic analysis was conducted to identify affected differentiated gene expression and signaling pathways. The molecular docking was performed to predict potential protein targets, and Western blot was used to validate expression and phosphorylation levels of key signaling molecules. The results demonstrated that 5Z-7-Oxozeaenol significantly suppressed proliferation and migration while promoting apoptosis in MDA-MB-231 cells. The transcriptomic analysis indicated enrichment in pathways related to cancer, cytokine–cytokine receptor interaction, MAPK and PI3K-Akt signaling, and cell adhesion molecules. The molecular docking suggested a high binding affinity between 5Z-7-Oxozeaenol and PTPRN. While Western blot analysis confirmed the downregulation of phosphorylated FAK, PI3K, Akt, and MAPK, along with reduced cyclin D1 expression. Additionally, 5Z-7-Oxozeaenol upregulated the pro-apoptotic proteins p53 and cleaved caspase-3. In conclusion, 5Z-7-Oxozeaenol exerts potent antitumor effects on MDA-MB-231 cells through multi-pathway inhibition and induction of apoptosis, highlighting its potential as a marine-derived therapeutic candidate for breast cancer treatment.

## 1. Introduction

Breast cancer remains the most frequently diagnosed malignancy and the leading cause of cancer-related mortality among women worldwide [1]. In recent years, both the incidence and detection of breast tumors have continued to rise, with the disease increasingly affecting younger populations, thus posing a major threat to women’s health [2]. According to the statistical analysis and release of the results of the International Agency for Research on Cancer (IARC) GLOBOCAN database in 2022, the incidence of breast cancer is expected to reach 11.6%, which is the highest among female malignant tumors worldwide, with a fatality rate of 6.9% [3]. Epidemiological surveys have shown that the disease burden of breast cancer is not optimistic in China, with the incidence of breast cancer being higher in urban areas than in rural areas [4]. Despite advances in medical standards around the world, the comprehensive treatment of breast cancer based on surgery has greatly extended the survival of breast cancer patients [1]; however, it is still an important public health problem worldwide because of its rapid progress and high mortality rate [5].

Unlike other breast cancer subtypes, triple-negative breast cancer (TNBC) lacks the expression of estrogen receptor (ER), progesterone receptor (PR), human epidermal growth factor receptor 2 (HER-2), making it unresponsive to hormonal and HER2-targeted therapies [6,7,8]. TNBC accounts for approximately 15–20% of all breast cancer cases and is associated with poor prognosis due to its aggressive nature and limited treatment options [2]. The heterogeneity of TNBC poses significant challenges in treatment, necessitating a broader screening of chemotherapeutic drugs [9,10,11,12].

5Z-7-Oxozeaenol has been reported to induce caspase-3-mediated apoptosis in HeLa cells and inhibit TGF-β-activated kinase 1 (TAK1) activity [13,14]. However, its biological activities and underlying mechanisms remain incompletely understood. In this study, we isolated 5Z-7-Oxozeaenol from the marine-derived fungus *Curvularia* sp. MDCW-1060, collected from the Beibu Gulf in Guangxi, China. We then performed a series of in vitro assays to evaluate its effects on breast cancer cells. Transcriptomic and bioinformatic analyses, combined with molecular biology approaches, were employed to elucidate the signaling pathways and molecular mechanisms through which 5Z-7-Oxozeaenol inhibits breast cancer cell proliferation. These findings provide a foundation for the development of novel therapeutic agents against breast tumors.

## 2. Results

### 2.1. 5Z-7-Oxozeaenol Structure

**Figure 1:**
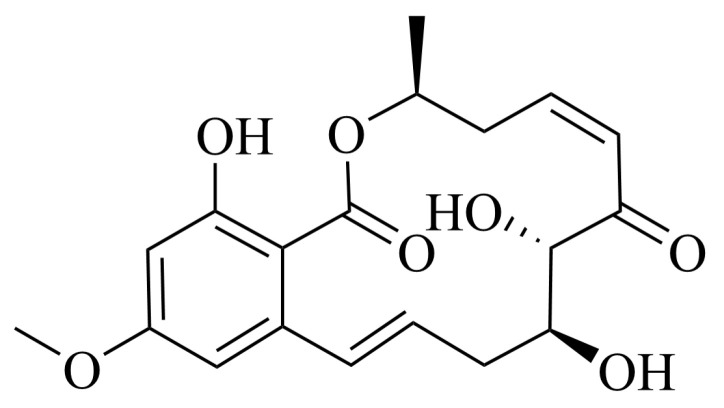
The structure of 5Z-7-Oxozeaenol.

Here, we report the isolation of 5Z-7-Oxozeaenol (C_19_H_22_O_7_), a fungal metabolite obtained from *Curvularia* sp. MDCW-1060—originally isolated from a crab specimen collected in the Beibu Gulf, Guangxi, China (Figure 1). The compound was obtained as a colorless powder, and its structure was elucidated through analysis of MS, ^1^H and ^13^C NMR spectral data (Figures S1–S3). The spectroscopic data were consistent with those previously reported for 5Z-7-Oxozeaenol [14], confirming its identity.

### 2.2. Antiproliferative Efficacy of 5Z-7-Oxozeaenol on Breast Cancer Cells

**Figure 2:**
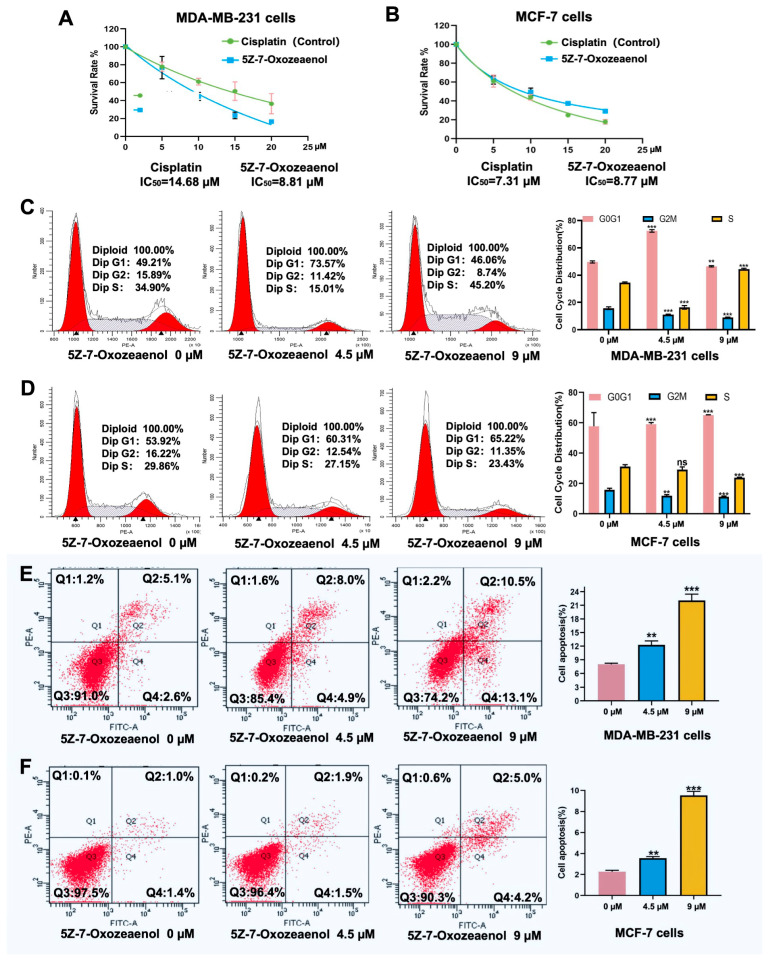
Antiproliferative efficacy of 5Z-7-Oxozeaenol on breast cancer cells: (**A**) The cell viability was determined by an MTT assay in MDA-MB-231 cells. (**B**) The cell viability was determined by a MTT assay in MCF-7 cells. (**C**) The cell cycle distribution was assessed by flow cytometric analysis in MDA-MB-231 cells. (**D**) The cell cycle distribution was assessed by flow cytometric analysis in MCF-7 cells. (**E**) The cell apoptosis rate was assessed by flow cytometric analysis in MDA-MB-231 cells. (**F**) The cell apoptosis rate was assessed by flow cytometric analysis in MCF-7 cells; ns, not significant, ** *p* < 0.01, *** *p* < 0.001, compared with the control group.

The antiproliferative activity of 5Z-7-Oxozeaenol was evaluated in MDA-MB-231 and MCF-7 breast cancer cells using the MTT assay, with cisplatin serving as a positive control. The results demonstrated that 5Z-7-Oxozeaenol suppressed cell proliferation in a dose-dependent manner. After 48 h of treatment with cisplatin and 5Z-7-Oxozeaenol in MDA-MB-231 cells, the half-maximal inhibitory concentration (IC_50_) values were 14.68 and 8.81, respectively. In the MCF-7 cells, the IC_50_ values were 7.30 and 8.77, correspondingly (Figure 2A).

Given the potent cytotoxicity of 5Z-7-Oxozeaenol, we further investigated its effects on cell cycle progression and apoptosis. Treatment of MDA-MB-231 and MCF-7 cells with increasing concentrations of 5Z-7-Oxozeaenol (0, 4.5, and 9 µM) for 48 h resulted in a concentration-dependent decrease in the proportion of cells in G2 phase, accompanied by an accumulation of cells in S and G1 phases (Figure 2B). These findings indicate that 5Z-7-Oxozeaenol induces cell cycle arrest primarily in S and G1 phases.

Apoptosis analysis revealed that 5Z-7-Oxozeaenol treatment significantly increased the total apoptotic cell population (including both early and late apoptotic cells) in MDA-MB-231 cells in a dose-dependent manner. The apoptotic rate increased from 7.7% (0 µM) to 12.9% (4.5 µM; *p* < 0.001) and further to 23.6% (9 µM; *p* < 0.001) (Figure 2B). Similarly, MCF-7 cells exhibited a concentration-dependent increase in apoptosis following 5Z-7-Oxozeaenol treatment (Figure 2C). Comparative analysis demonstrated that MDA-MB-231 cells showed significantly greater sensitivity to 5Z-7-Oxozeaenol-induced apoptosis compared to MCF-7 cells (Figure 2C). Notably, 5Z-7-Oxozeaenol exhibited superior efficacy in both suppressing proliferation and inducing apoptosis in MDA-MB-231 cells when compared to the conventional chemotherapeutic agent cisplatin. Based on these findings, subsequent mechanistic studies were conducted using MDA-MB-231 cells.

### 2.3. 5Z-7-Oxozeaenol Inhibited MDA-MB-231 Cells Migration and Growth

**Figure 3:**
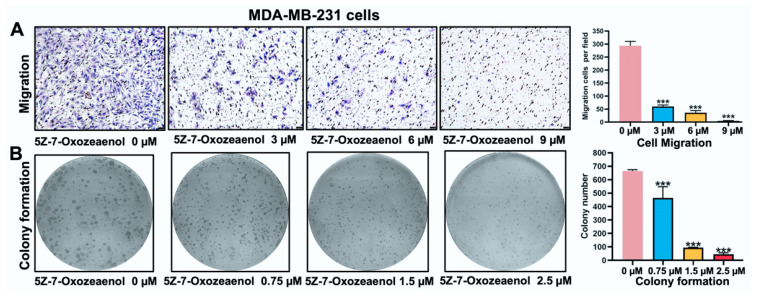
5Z-7-Oxozeaenol inhibited MDA-MB-231 cells migration and growth. (**A**) Transwell assays evaluating the migration ability of MDA-MB-231 cells treated with the indicated concentrations of 5Z-7-Oxozeaenol for 48 h. (**B**) MDA-MB-231 cells were treated with the indicated concentrations of 5Z-7-Oxozeaenol for 8 days, after which colony formation was assessed; *** *p* < 0.001, compared with the control group.

Transwell assays were used to examine the effects of 5Z-7-Oxozeaenol on cell migration ability. Treatment with 5Z-7-Oxozeaenol resulted in a significant reduction in the number of migrated cells compared to the control group, demonstrating its potent inhibitory effect on MDA-MB-231 cell migration (Figure 3A). Furthermore, colony formation assays revealed that 5Z-7-Oxozeaenol suppressed cell growth in a concentration-dependent manner, confirming its strong anti-proliferative activity (Figure 3B).

### 2.4. RNA-Seq Analysis of MDA-MB-231 Cells Treated with 5Z-7-Oxozeaenol

**Figure 4:**
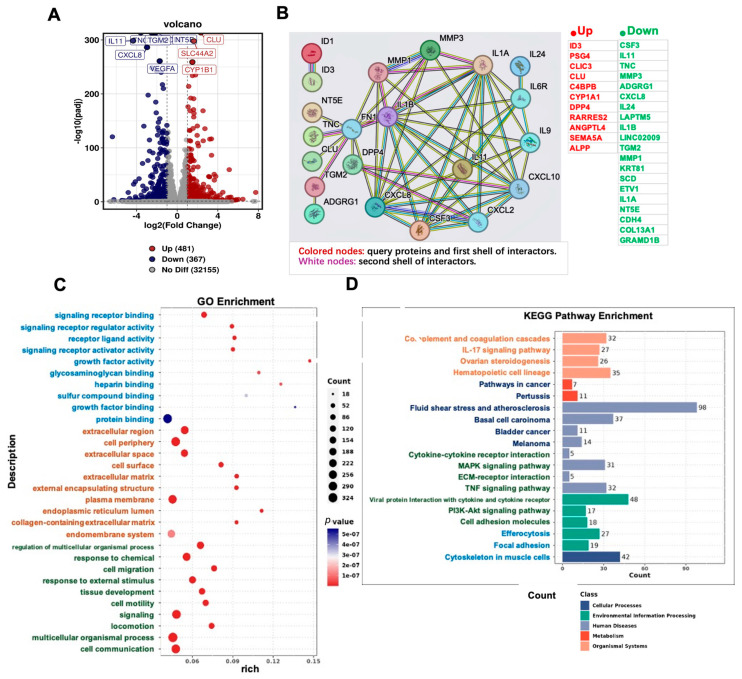
Transcriptome analysis of MDA-MB-231 cells treated with compound 5Z-7-Oxozeaenol vs. the control: (**A**) Volcanic map showing the differential gene expression distribution. The scattered dots in the figure represent individual genes, with grey dots indicating genes whose expression did not with not significantly differ, red dots representing upregulated genes whose expression did not significantly increased, and blue dots representing downregulated genes whose expression significantly decreased. (**B**) Construction of the PPI network for the identification of hub genes. Colored nodes: query proteins and first shell of interactors. White nodes: second shell of interactors. The red bars on the right side of the PPI chart represent: 11 up-regulated genes with |log_2_(FoldChange)| > 2.0 were selected from the DEGs. The green bars on the right side of the PPI chart represent: 20 down-regulated genes with |log_2_(FoldChange)| > 2.0 were identified from the DEGs. (**C**) GO functional enrichment analysis of the DEGs. (**D**) KEGG pathway enrichment of DEGs.

To elucidate the mechanism of action of 5Z-7-Oxozeaenol on MDA-MB-231 cells, RNA-seq was performed. Transcriptomic analysis following treatment with 5Z-7-Oxozeaenol (9.0 μM) for 48 h revealed distinct clustering of gene expression profiles between treated and control groups, as visualized in the heatmap (Figure 4A).

The RNA-seq identified 1966 differentially expressed genes (DEGs), comprising 1063 significantly upregulated and 903 downregulated genes (Figure 4A). A total of 20 down-regulated genes with |log_2_(FoldChange)| > 2.0 were identified from the DEGs: CSF3, IL11, TNC, MMP3, ADGRG1, CXCL8, IL24, LAPTM5, IL1B, LINC02009, TGM2, MMP1, KRT81, SCD, ETV1, IL1A, NT5E, CDH4, COL13A1, and GRAMD1B (indicated in the green bar on the right side of Figure 4B). Additionally, 11 up-regulated genes that met the same fold change threshold were selected: ID3, PSG4, CLIC3, CLU, C4BPB, CYP1A1, DPP4, RARRES2, ANGPTL4, SEMA5A, and ALPP (shown in the red bar on the right side of Figure 4B). These genes were subsequently used to construct a protein–protein interaction (PPI) network using the STRING database. PPI network analysis revealed that 5Z-7-Oxozeaenol markedly altered the expression of extracellular matrix (ECM)-related genes, including MMP subfamily members (MMP1, MMP3), fibronectin (FN) and its regulatory genes (NT5E, TNC, CLU, TGM2), CXC chemokine genes (CXCL2, CXCL8, CXCL10), and immunoregulatory genes (IL1A, IL1B, IL11, IL6R, IL9, IL24), all of which were significantly downregulated after 5Z-7-Oxozeaenol treatment (Figure 4B).

Gene Ontology (GO) enrichment analysis highlighted the overall functional characteristics of DEGs, which are significantly enriched genes related to core biological functions, including biological process (BP), molecular function (MF), and cellular component (CC). The enrichment of the results indicated that the response to stimulation, multicellular organism processes, developmental processes, positive regulation of biological processes, and anatomical structure development were clustered in the top five positions of the enriched BP terms. The cell periphery, plasma membrane, endomembrane system, extracellular region and vesicle were clustered in the top five positions of the CC terms. Binding, protein binding, molecular function regulator activity, enzyme binding and signaling receptor binding were clustered in the top five positions of the MF terms (Figure 4C). The Gene Set Enrichment Analysis (GSEA) further supported these findings, indicating significant enrichment in protein tyrosine kinase binding, regulation of immune system processes, peptidase activity, xenobiotic metabolic process, and progesterone metabolic process (Figure S4).

The Kyoto Encyclopedia of Genes and Genomes (KEGG) pathway analysis was conducted to identify the pathways involved. The enrichment results for the DEGs related to functional pathways indicated that 5Z-7-Oxozeaenol had a significant effect on human diseases, organismal systems, and environmental information processing (Figure 4D). Multiple pathways with a high number of DEGs included pathways related to cancer, cytokine–cytokine receptor interaction, MAPK signaling pathway, PI3K-Akt signaling pathway, and cell adhesion molecules. These pathways form an interactive network, with cancer pathways exhibiting notable crosstalk with both PI3K-Akt and MAPK signaling cascades, collectively regulating critical cellular behaviors such as proliferation and apoptosis.

### 2.5. Molecular Docking for 5Z-7-Oxozeaenol with the Protein Tyrosine Phosphatase Receptor Type N (PTPRN)

**Figure 5:**
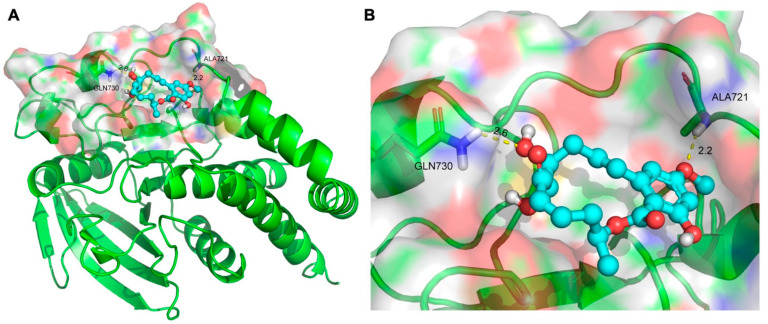
The binding mode of 5Z-7-Oxozeaenol to its targets, determined by molecular docking: (**A**) Three-dimensional structures of the binding pockets were constructed for the binding mode of 5Z-7-Oxozeaenol to PTPRN (PDB: 2I1Y): (**B**) 5Z-7-Oxozeaenol docking with the active site of the target protein PTPRN.

To assess the binding affinity of 5Z-7-Oxozeaenol with potential targets, molecular docking analysis was performed, identifying the receptor-type protein tyrosine phosphatase PTPRN (PDB: 2I1Y) as the top-ranking candidate. The docking results demonstrated that 5Z-7-Oxozeaenol forms hydrogen bonds with key residues ALA721 and GLN730 in the active site of PTPRN, accompanied by a favorable binding energy of −7.6 kcal/mol, indicating a highly stable interaction (Figure 5A,B). These findings suggest that 5Z-7-Oxozeaenol may exert its biological effects by binding to and modulating the activity of PTPRN. Functionally, PTPRN acts as a phosphatase that dephosphorylates tyrosine residues on downstream signaling proteins—including receptor tyrosine kinases (RTKs) and focal adhesion kinase (FAK)—thereby attenuating proliferation, survival, and migration signals mediated by tyrosine phosphorylation.

### 2.6. Western Blot Analysis of Relative FAK, PI3K-Akt, MAPK Protein Expression

**Figure 6:**
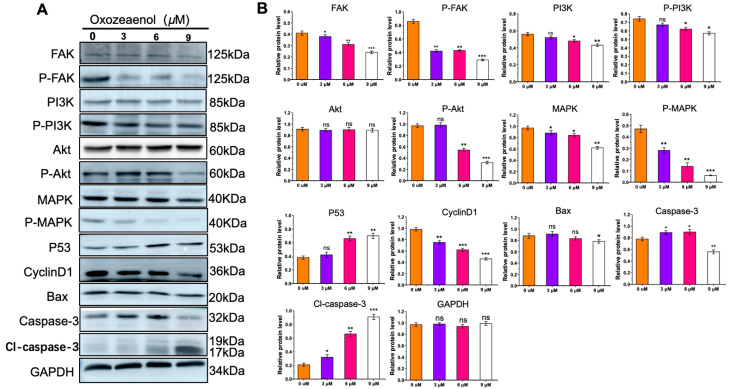
Effects of 5Z-7-Oxozeaenol on FAK, PI3K-Akt and MAPK pathway-related protein expression in MDA-MB-231 cells. (**A**) FAK/p-FAK, PI3K/p-PI3K, Akt/p-Akt, MAPK/P-MAPK, p53, Cyclin D1, Bax, Caspase-3 and Cl-caspase-3 protein levels were analyzed by Western blotting. The analysis utilized actin as a reference for quantifying relative protein expression levels. (**B**) Relative quantification of protein expression compared with that in the control group. ns, not significant, * *p* < 0.05, ** *p* < 0.01, and *** *p* < 0.001, compared with the control group.

To further validate the KEGG and molecular docking predictions, we examined the expression and phosphorylation levels of key signaling molecules—FAK, PI3K-Akt, and MAPK—implicated in cell proliferation via Western blotting. Treatment of MDA-MB-231 cells with increasing concentrations of 5Z-7-Oxozeaenol for 6 h resulted in a dose-dependent reduction in the phosphorylation levels of FAK, PI3K-Akt, and MAPK (Figure 6A,B). Concurrently, the protein expression of the tumor suppressor p53 increased, while levels of the downstream effector Cyclin D1 decreased in a dose-dependently. Furthermore, a marked increase in cleaved caspase-3 (Cl-caspase-3) was observed following treatment. These results indicate that 5Z-7-Oxozeaenol suppresses cell proliferation and induces apoptosis likely through inhibition of FAK and its downstream PI3K-Akt and MAPK signaling pathways.

## 3. Discussion

The Beibu Gulf, a semi-enclosed bay situated in the northwestern South China Sea [15], is recognized as a region of global biodiversity significance [16]. It hosts representative marine ecosystems—such as coral reefs, mangroves, seagrass beds, and coastal wetlands—that support rich microbial and broader biological diversity [17]. Between November 2003 and September 2022, scientific studies have reported the discovery of 477 marine natural products from this region, among which 206 are marine terpenoids [18]. Of these terpenoids, 72 have demonstrated notable bioactivities, including cytotoxicity (24 compounds), antibacterial effects (19 compounds), enzyme inhibition (13 compounds), anti-inflammatory properties (10 compounds), among others [19]. Marine natural products play a vital role in the discovery and development of novel therapeutics [18]. Given their significant biological activities and therapeutic potential against various human diseases, these compounds have attracted growing interest among biochemists and pharmacologists [20,21].

5Z-7-Oxozeaenol, originally isolated from a Phoma-related fungal strain (MSX 63935; Mycosynthetix library), was initially identified as a fungal metabolite with limited bioactivity. However, it has since been recognized as a highly selective and potent kinase inhibitor, exhibiting notable antiproliferative and anti-inflammatory properties [14]. Previous studies demonstrated that combining 5Z-7-Oxozeaenol with hyperthermia (HT) enhanced cell death in KRAS-mutant A549 lung cancer cells in a dose-dependent manner [22]. This effect was accompanied by increased reactive oxygen species (ROS) production, loss of mitochondrial membrane potential, and elevated expression of caspase-3 and -7 in HeLa cells, compared to paclitaxel treatment [23]. Additionally, 5Z-7-Oxozeaenol acts as an effective inhibitor of the NF-κB pathway and induces caspase-3 and -7 expression in both HeLa and HT-29 cancer cells [24,25].

In the present study, we investigated the molecular mechanisms underlying the inhibitory effects of 5Z-7-Oxozeaenol in triple-negative breast cancer MDA-MB-231 cells. Our results demonstrate that treatment led to a significant G1 phase cell cycle arrest, accompanied by concentration-dependent suppression of migration and colony formation.

KEGG pathway analysis suggested that the antiproliferative effects of 5Z-7-Oxozeaenol involve multiple interconnected signaling pathways, including FAK, PI3K-Akt, and MAPK, which collectively regulate cellular behaviors. The FAK pathway exhibits extensive crosstalk with both PI3K-Akt and MAPK cascades [26]. For instance, the FAK–Src complex directly phosphorylates and activates PI3K [27], leading to PIP_3_ production and subsequent Akt activation—a key mechanism promoting cell survival, proliferation, and migration [28]. Furthermore, FAK–Src activates Ras, initiating the Raf–MEK–ERK cascade to drive proliferation and differentiation [29]. FAK also upregulates matrix metalloproteinases (MMPs) via MAPK/ERK or PI3K/AKT signaling, facilitating cell invasion [30,31,32,33]. The PI3K-Akt and MAPK signaling pathways engage in extensive crosstalk, forming a complex, interconnected network that co-regulates essential cellular processes such as proliferation, survival, differentiation, and metabolism. Additionally, Akt can phosphorylate and modulate components of the MAPK pathway, including RAF and MEK, thereby fine-tuning MAPK signaling activity. Conversely, RSK, a downstream effector of MAPK, can phosphorylate and inhibit upstream elements of the PI3K-Akt pathway, creating negative feedback loops. Such reciprocal regulation ensures signal specificity, amplification, and homeostasis [34].

The Western blot analysis confirmed that 5Z-7-Oxozeaenol dose-dependently reduced phosphorylation levels of FAK, PI3K, Akt, and MAPK. Since Akt activation suppresses apoptosis by phosphorylating caspase-9 and inhibiting caspase-3 cleavage, as well as downregulating p53 and Bax, our findings that 5Z-7-Oxozeaenol decreased p-Akt levels while increasing p53, Bax, and cleaved caspase-3 are consistent with Akt pathway inhibition and subsequent induction of MDA-MB-231 cells apoptosis and cell cycle arrest.

Moreover, PPI analysis indicated that 5Z-7-Oxozeaenol downregulated expression of several extracellular matrix (ECM)-related genes (NT5E, TNC, CLU, TGM2) and MMPs (MMP1, MMP3), likely resulting from suppressed FAK phosphorylation.

Cyclin D1, a critical regulator of G1–S transition, is transcriptionally regulated by both MAPK and PI3K/Akt pathways [35,36,37]. Conversely, p53 activation induces CDK inhibitors such as p21, thereby inhibiting Cyclin D1–CDK4/6 activity [34]. In line with this, 5Z-7-Oxozeaenol treatment significantly reduced Cyclin D1 expression in MDA-MB-231 cells, providing a mechanistic basis for the observed G1 arrest.

Through target screening, PTPRN emerged as a top candidate target of 5Z-7-Oxozeaenol. PTPRN, a receptor-type protein tyrosine phosphatase, plays a regulatory role in both the MEK/ERK and PI3K/AKT signaling pathways, contributing to the complex cross-talk between these critical cascades [38]. PTPRN was upregulated in lung adenocarcinoma (LUAD) and promotes metastasis and poor prognosis [39]. In colorectal cancer, PTPRN deletion impairs invasiveness and metastatic potential through dysregulated epithelial–mesenchymal transition (EMT) and insulin receptor signaling [40]. Based on molecular docking and Western blot results, we propose that 5Z-7-Oxozeaenol binds to PTPRN, perturbing its catalytic function and modulating downstream FAK and PI3K-Akt pathways, ultimately impairing cell proliferation, survival, and migration. Nonetheless, the involvement of additional signaling pathways cannot be excluded and warrants further investigation.

In summary, 5Z-7-Oxozeaenol exerts significant anti-tumor effects on MDA-MB-231 cells by inducing G1 phase cell cycle arrest, suppressing migration and colony formation, and promoting apoptosis. Mechanistically, it inhibits phosphorylation of key signaling nodes within the FAK/PI3K-Akt/MAPK network, leading to reduced Akt activation, increased expression of pro-apoptotic proteins (p53, Bax, and cleaved caspase-3), and downregulation of Cyclin D1. Additionally, PPI revealed that 5Z-7-Oxozeaenol downregulated extracellular matrix-related genes and MMPs likely through FAK suppression. Preliminary target screening suggests PTPRN as a potential binding partner, through which it may modulate downstream pathways to impair malignant phenotypes, though further validation is required to elucidate full mechanistic breadth.

## 4. Materials and Methods

### 4.1. Fungal Materials

*Curvularia* sp. MDCW-1060 was isolated from a crab sample collected at the Guangxi Beibu Gulf and identified by the 18S rDNA gene sequence (GenBank accession no. PV355094).

### 4.2. Fermentation and Extraction

*Curvularia* sp. MDCW-1060 was cultured at 28 °C for 4 days on PDA medium. Spores were cultivated in 60 × 1000 mL Erlenmeyer flasks (120 mL seawater, 80 g rice). The flasks were cultured under static conditions for 30 days at room temperature. The culture was extracted with EtOAc to yield 52.0 g.

### 4.3. Isolation

The extracts were further extracted by 90% MeOH–H_2_O and petroleum ether, yielding 41.5 g of petroleum ether extract and 9.4 g of MeOH–H_2_O extract. The methanol layer extract was fractionated into 8 fractions (Fr.1–Fr.8) by VLC and eluting with petroleum and ether–EtOAc. Fr.7 (420.2 mg) was purified by HPLC on an ODS column (from 50:50 to 85:15 MeOH/H_2_O) to yield compound 5Z-7-Oxozeaenol (29.0 mg, *t*_R_ = 11.6 min). 5Z-7-Oxozeaenol is a colorless powder and was isolated, and the following experimental procedures were performed: Preparative HPLC was performed on an LC3000 system using a preparative C_18_ column (5 µm, 20 × 250 mm, 10 mL/min Cosmosil, Kyoto, Japan). Analytical HPLC was performed on a Shimadzu LC-10AD VP system using an analytical HPLC C_18_ column (5 µm, 4.6 × 250 mm, 1 mL/min Cosmosil, Kyoto, Japan). Optical rotation was measured on SGWzz-1 digital polarimeter, HRESIMS spectra were obtained using a Mariner API-TOF, and NMR spectra were recorded on a Bruker Avance 400 spectrometer (Bruker, Fallanden, Switzerland). The optical rotation for 5Z-7-Oxozeaenol is ${\left[\alpha \right]}_{D\;}^{25}$ = −148.3 (c = 0.12, MeOH). The structure of 5Z-7-oxozeaenol was determined by its MS, ^1^H and ^13^C NMR data (Table 1).

### 4.4. Cell Culture and Reagents

The human cell lines MDA-MB-231 and MCF-7 were purchased from the Institute of Biochemistry and Cell Biology, Chinese Academy of Sciences (Shanghai, China). Cells were cultured in Dulbecco’s Modified Eagle Medium (DMEM) supplemented with 10% fetal bovine serum (FBS) and 1% penicillin–streptomycin in a humidified incubator at 37 °C with 5% CO_2_. Stock solutions of 5Z-7-Oxozeaenol were dissolved in dimethylsulfoxide (DMSO), and all treated concentrations were adjusted in the culture medium.

### 4.5. Cell Proliferation Assays

An MTT assay was used to assess cell viability following exposure to 5Z-7-Oxozeaenol. Cells were initially seeded at a density of 1 × 10^4^ per well in 96-well plates and cultured overnight. In the MTT experiment, five distinct groups of cells were treated with 5Z-7-Oxozeaenol (0, 5, 10, 15, and 20 µM) at 37 °C for 48 h, with each concentration having four replicates. The plates were then added with 0.5 mg/mL MTT solution and incubated at 37 °C for another 4 h. The supernatant was separated, and the formazan crystals were dissolved in 1 mL of DMSO. An aliquot of the DMSO lysed solution (200 μL) was obtained from the 24-well plates and transmitted to 96-well reader plates. The absorbance was measured at a wavelength of 490 nm using a microplate reader (Bio Tek Instruments, Bad Friedrichshall, Germany). The IC_50_ values were calculated using GraphPad Prism 9.5.

### 4.6. Cell Cycle Assay

MDA-MB-231 cells were cultured in a 6-well plate at a density of 2 × 10^5^ cells per well and treated with 5Z-7-Oxozeaenol at concentrations of 0, 4.5, and 9 µM for a duration of 48 h. Following treatment, the MDA-MB-231 cells and MCF-7 cells were harvested and fixed overnight using ice-cold ethanol (75%). A working solution containing propidium iodide (PI) at a concentration of 100 μg/mL and RNase A at the same concentration was subsequently added to each sample (500 μL). The samples were then incubated in the dark for 30 min. The cell cycle distribution was analyzed via flow cytometry using an LSRFortessa instrument from BD Biosciences (Canton, MA, USA). For data analysis related to cell cycle progression, ModFit LT version 5.0 software was employed. This experiment was conducted in triplicate to ensure reproducibility.

### 4.7. Apoptosis Assays

To assess apoptosis, MDA-MB-231 cells and MCF-7 cells were seeded at a density of 3 × 10^5^ cells per well in 6-well culture plates and incubated for 24 h. Following this incubation period, the cells were treated with different concentrations 5Z-7-Oxozeaenol. After an additional two days of incubation, the cells were harvested and resuspended in 1× Annexin-binding buffer. They were subsequently stained with FITC-conjugated Annexin V and propidium iodide (PI). Data acquisition and analysis were performed using a flow cytometer, ensuring that data from a minimum of 10,000 cells per sample were collected for accurate results.

### 4.8. Cell Migration Assays

The experimental protocol for assessing cellular migration was conducted as below: First, MDA-MB-231 cells were subjected to serum starvation for 12 h to achieve cell cycle synchronization, followed by trypsinization and resuspension in serum-deprived medium. For the cell migration assay, 1 × 10^5^ cells were seeded into upper chambers with serum-free medium, while the lower chambers contained medium with 20% FBS as a chemoattractant in Transwell system equipped with an 8-μm porous membrane (Labselect, Hangzhou, China). After cells were cultured at 37 °C with 5% CO_2_ for 48 h, the migrated cells adhering to the underside of the membrane were fixed using 4% paraformaldehyde (Biosharp, Shanghai, China) for 30 min and subsequently visualized with 0.5% crystal violet staining for 15 min. Nonmigratory cells remaining on the upper membrane surface were removed using cotton swabs. Quantitative analysis was performed by counting violet-stained cells in three randomly chosen microscopic fields at 100× magnification, with representative images documented using an inverted phase-contrast microscope.

### 4.9. Cell Colony Formation Assay

In the colony formation assay, MDA-MB-231 cells were seeded in 6-well plates at a density of 500 cells per well and cultured for 24 h. Then, after treating the cells with 5Z-7-oxozeaenol, cells were continuously cultured for 8 days. The growth medium was replaced every 72 h. When colony formation was clearly observed under a microscope, the culture medium was aspirated, and the cells were fixed with 4% paraformaldehyde solution for 15 min. The plate was rinsed three times with phosphate-buffered saline (PBS), and then stained with 0.5% crystal violet solution for 15 min. Subsequently, the plate was washed with PBS to remove unbound dye, ensuring optimal visualization and quantification of colonies.

### 4.10. RNA-Seq

Total RNA was extracted from cells using a TRIzol reagent kit (Invitrogen, Carlsbad, CA, USA) and assessed for quality using an Agilent 2100 Bioanalyzer system (Agilent Technologies, Palo Alto, CA, USA). Library preparation for sequencing was subsequently conducted using the VAHTS Universal V6 RNA-seq Library Prep Kit for MGI (Vazyme, Nanjing, China) and sequencing was performed on the DNBSEQ T7 platform (Benagen, Wuhan, China). The obtained clean data were processed by eliminating reads containing adapters and low-quality sequences from the raw data. Alignments of the clean reads with the assembled genome of GRCh38_Ensembl_114 were performed using STAR (2.7.9a). The FPKM value of each gene in each sample was determined using RSEM (v 1.3.3). Genes with a *p*-value of <0.05 and a fold change of >2 were considered to be significantly differentially expressed.

### 4.11. Construction of the Protein—Protein Interaction (PPI) Network

A protein—protein interaction (PPI) network was constructed using the STRING (https://cn.string-db.org) database, with screening parameters set to a confidence score of 0.7. The cytoHubba plug-in within the Cytoscape 3.10.4 software was used to identify the core modules and the key gene targets with the top 20 scores within the PPI network.

### 4.12. GO and KEGG Pathway Enrichment Analysis

An enrichment analysis of GO and KEGG pathway terms was subsequently conducted with specific parameters (enrichment conditions: overlap ≥ 3, *p* value cut-off ≤ 0.05). For the GO enrichment analysis, the 10 pathways with the highest count values and *p* < 0.01 were selected, encompassing cellular components, molecular functions, and biological processes. Visualization and analysis were carried out using a bioinformatics platform (http://www.bioinformatics.com.cn/, accessed on 16 August 2025) to generate Sankey diagrams and histograms.

### 4.13. Molecular Docking

A computer simulation approach was employed to evaluate 5Z-7-Oxozeaenol through molecular docking with target proteins. The Maestro program in Glide software version 8.1 was used for docking. The human target protein human PTPRN (ID:2I1Y) was obtained from the PDB protein database (15 September 2025), while the structures of the 5Z-7-oxozeaenol compounds were downloaded from the PubChem database (15 September 2025) and obtained from the PDB database (http://www.rcsb.org, accessed on 15 September 2025).

Prior to docking, water molecules were eliminated from the 3D conformation, and the proteins were hydrogenated. The 3D structures of the target compounds were subsequently energetically minimized for optimization. Molecular docking analysis was conducted to assess the binding interactions between the ligands and receptors, and the potential active compounds were evaluated on the basis of the -CDOCKER ENERGY score.

### 4.14. Western Blot Analysis

MDA-MB-231 cells were planted in 6 cm dishes at a density of 5 × 10^5^ cells/dish for 24 h and then cultured with various drugs as explained in figure legends. Cells were collected as programmed after treatment under various conditions. Determine protein concentration using the BCA method. Equivalent total proteins (20 μg) of cell lysates were separated through 10% SDS-PAGE and then transferred onto a PVDF membrane for 50–75 min. The PVDF membranes were maintained with 5% nonfat milk in PBST buffer for 1 h to block nonspecific binding. After blocking, the PVDF membranes were incubated with the following primary antibodies at 4 °C overnight. Afterwards, the HRP-conjugated goat anti-mouse (1:3000) or -rabbit secondary antibody (1:3000) was added, and the samples were incubated at room temperature for 2 h. Immunoreactive bands were analyzed using a chemiluminescence Western blot detection system, and quantification was performed using ImageJ 1.8.0 software. All primary antibodies were purchased from Cell Signaling Technology (“CST”, Danvers, MA, USA) (Table 2), and secondary antibodies were purchased from Affinity Biosciences.

### 4.15. Statistical Analysis

We utilized Prism GraphPad 9.5 software to perform all the statistical analyses, including Student’s *t*-test, one-way ANOVA, and two-way ANOVA; * *p* <0.05, ** *p* < 0.01, *** *p* < 0.001, **** *p* < 0.0001.

## Figures and Tables

**Table 1:** ^1^H-NMR (400 MHz) and ^13^C-NMR (100 MHz) data of 5Z-7-Oxozeaenol in DMSO-*d*_6_.

No.	*δ*_C_, Type	*δ*_H_, Mult. (*J* in Hz)
1	170.4 (C)	
3	73.8 (CH)	5.20, qdd (12.0, 5.9, 1.9)
4	36.3 (CH2)	2.58, m
		3.20, m
5	142.4 (CH)	6.19, ddd (13.6, 10.0, 3.5)
6	126.7 (CH)	6.43, dd (11.3, 2.6)
7	200.9 (C)	
8	81.7 (CH)	4.38, m
9	72.7 (CH)	3.85, m
10	36.0 (CH2)	2.02, m
11	132.4 (CH)	6.06, ddd (14.9, 9.2, 5.2)
12	131.0 (CH)	6.74, dd (15.3)
13	143.9 (C)	
14	106.1 (CH)	6.42, d (2.5)
15	163.4 (C)	
16	100.2 (CH)	6.38, d (2.5)
17	163.7 (C)	
18	104.5 (C)	
19	20.1 (CH3)	1.37, d (6.1)
20	55.5 (CH3)	3.78, s
8-OH		4.98, d (4.9)
9-OH		5.06, d (6.1)
17-OH		11.77, s

**Table 2:** The primary antibody product used in this article.

No.	Primary Antibody	Dilution	Isotype	Source
1	FAK	1:1000	Rabbit IgG (#71433)	Cell Signaling Technology
3	P-FAK	1:1000	Rabbit IgG (#8556)	Cell Signaling Technology
4	PI3K	1:1000	Rabbit IgG (#4257)	Cell Signaling Technology
5	P-PI3K	1:1000	Tyr458/199 (#4228)	Cell Signaling Technology
6	Akt	1:1000	Rabbit IgG (#4691)	Cell Signaling Technology
7	P-Akt	1:2000	Rabbit IgG (#4060)	Cell Signaling Technology
8	MAPK	1:1000	Rabbit IgG (#8690)	Cell Signaling Technology
9	P-MAPK	1:1000	Rabbit IgG (#4377)	Cell Signaling Technology
10	P53	1:1000	Rabbit IgG (#2527)	Cell Signaling Technology
11	CyclinD1	1:1000	Rabbit IgG (#55506)	Cell Signaling Technology
12	Bax	1:1000	Rabbit IgG (#5023)	Cell Signaling Technology
13	Caspase-3	1:1000	Rabbit IgG (#14220)	Cell Signaling Technology
14	Cleaved-caspase-3	1:1000	Rabbit IgG (#9664)	Cell Signaling Technology
15	GAPDH	1:1000	Mouse IgG (#T0004)	Cell Signaling Technology

## Data Availability

The data presented in this study are available upon request from the corresponding author.

## Supplementary Materials

The following supporting information can be downloaded at: https://www.mdpi.com/article/10.3390/md23110414/s1, Figure S1: ^1^H NMR (400 MHz) spectrum of compound **1** in DMSO-*d*_6_. Figure S2: 13C NMR (100 MHz) spectrum of compound 1 in DMSO-*d*_6_. Figure S3: HR-ESI-MS spectrum of compound **1**. Figure S4: GSEA was conducted using GO pathways’ biological process branch as the gene sets of interest.

## Abbreviations

The following abbreviations are used in this manuscript:

IARC	International agency for research on cancer
PI	Propidium iodide
PTPRN	Protein tyrosine phosphatase receptor type N
MTT	Methylthiazolyldiphenyl-tetrazolium bromide
DEGs	Differentially expressed genes
PPI	Protein–Protein interactions
GO	Gene ontology
KEGG	Kyoto encyclopedia of genes and genomes
GSEA	Gene set enrichment analysis
BP	Biological Processes
MF	Molecular functions
CC	Cellular components
MMP	Matrix metallopeptidase
RTKs	Receptor tyrosine kinases
FAK	Focal adhesion kinase
ECM	Extracellular Matrix
HT	Hyperthermia
ROS	Reactive oxygen species
PFs	Purkinje Fibers
PTP	Protein tyrosine phosphatase
LUAD	Lung adenocarcinoma
CRC	Colorectal cancer
