# Supplementary material for: 5Z-7-Oxozeanol Isolated from the Fungus Curvularia sp. MDCW-1060 Inhibits the Proliferation of MDA-MB-231 Cells via the PI3K-Akt and MAPK Pathways
Source: Mar Drugs. 2025 Oct 23;23(11):414. doi: 10.3390/md23110414 (PMC12653621; doi:10.3390/md23110414)
Supplement: Supplementary file 1 [file marinedrugs-23-00414-s001.zip › marinedrugs-3931864-supplementary.pdf]

Supplementary Information

5Z-7-Oxozeanol Isolated from the Fungus *Curvularia* sp. MDCW-1060 Inhibits the Proliferation of MDA-MB-231 Cells via the PI3K-Akt and MAPK Pathways

Hong Zhang, Jianjian Wang, Chang Xu, Kai Liu, Jufang Xie, Zhoucheng He, Yonghong Liu, Cong Wang and Xinjian Qu

List of Supporting Information

Figure S1. ^{1}H NMR (400 MHz) spectrum of compound **1** in DMSO-d_6

Figure S2. ^{13}C NMR (100 MHz) spectrum of compound **1** in DMSO-d_6

Figure S3. HR-ESI-MS spectrum of compound **1**

Figure S4. GSEA was conducted using GO pathways' biological process branch as the gene sets of interest.

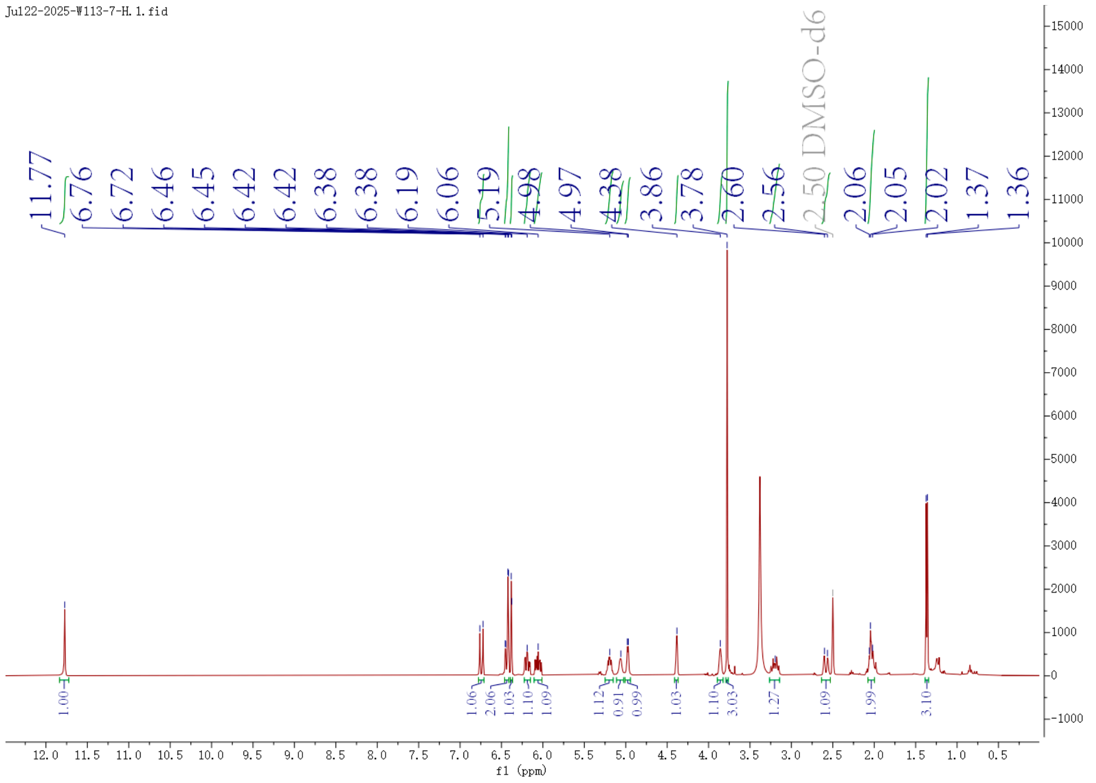

Figure S1. ^{1}H NMR (400 MHz) spectrum of compound **1** in DMSO-d_6

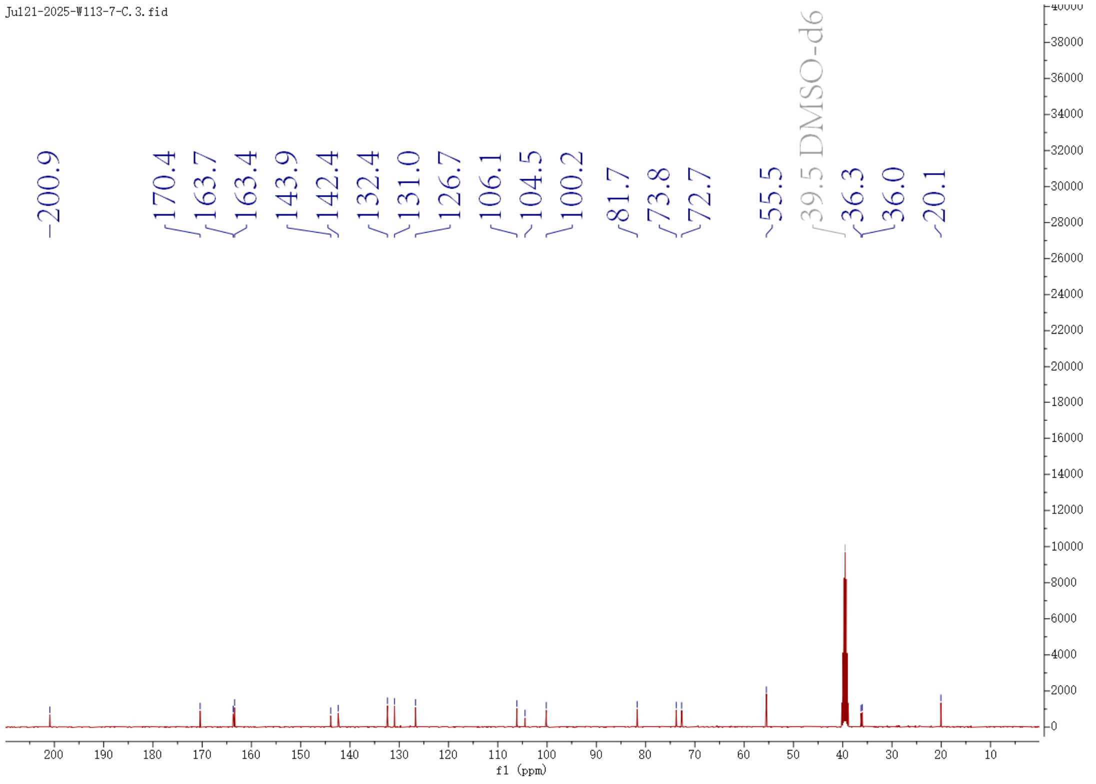

Figure S2. ^{13}C NMR (100 MHz) spectrum of compound **1** in DMSO-d_6

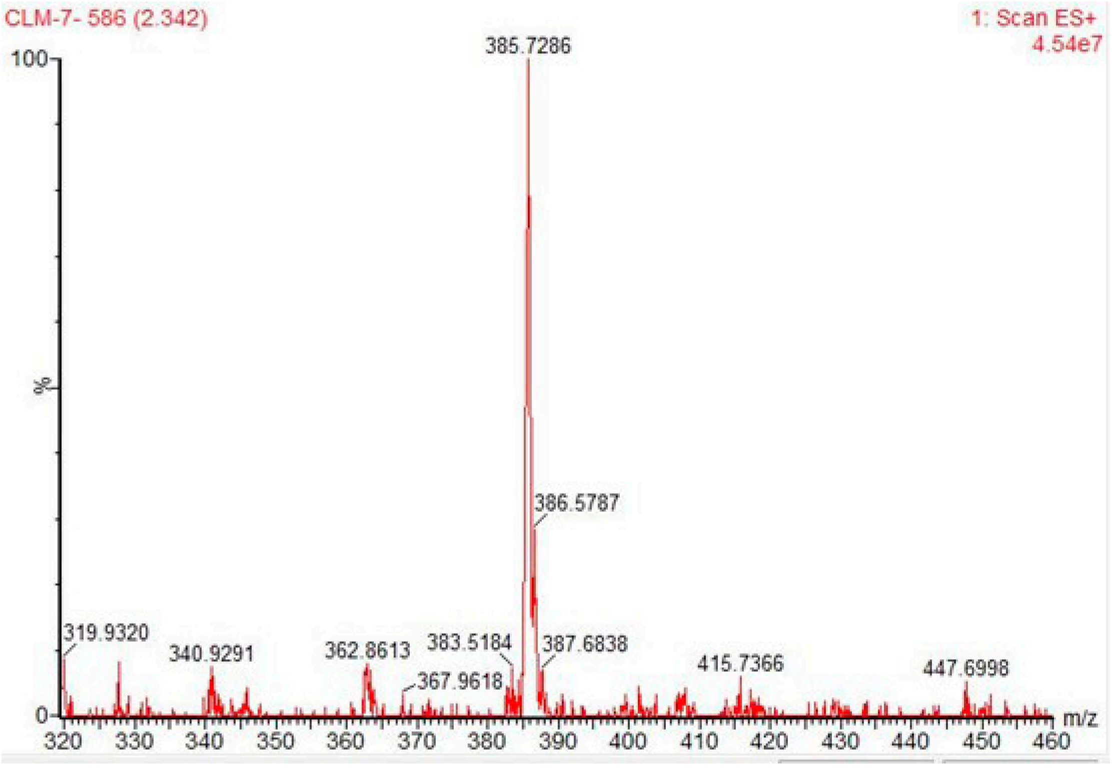

Figure S3. HR-ESI-MS spectrum of compound **1**

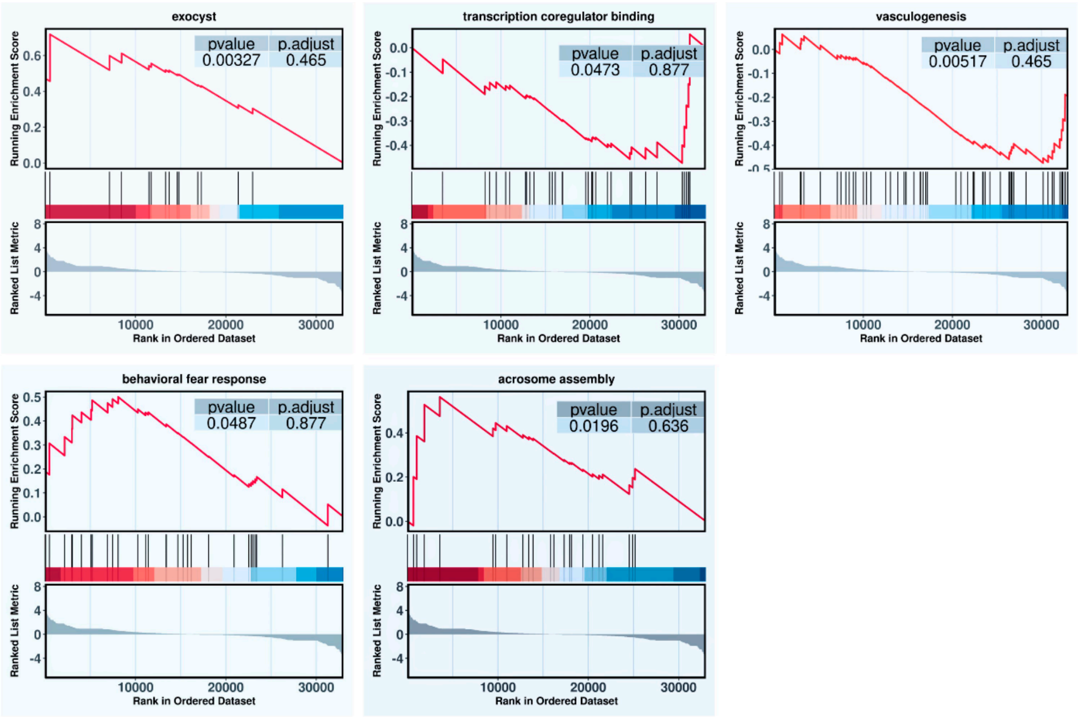

Figure S4. GSEA was conducted using GO pathways' biological process branch as the gene sets of interest.
